# Effect of Daily *Lactococcus cremoris* spp. Consumption Immobilized on Oat Flakes on Blood and Urine Biomarkers: A Randomized Placebo-Controlled Clinical Trial

**DOI:** 10.3390/medicina61060956

**Published:** 2025-05-22

**Authors:** Panoraia Bousdouni, Aikaterini Kandyliari, Anastasia Kargadouri, Panagiota Potsaki, Olga I. Papagianni, Maria-Eleni Stylianou, Nikoletta Stathopoulou, Panagiota Andrianopoulou, Maria Kapsokefalou, Vasiliki Bountziouka, Anastasia Kolomvotsou, Ioanna Prapa, Gregoria Mitropoulou, Chrysoula Pavlatou, Andreas G. Tzakos, Panayiotis Panas, Yiannis Kourkoutas, Antonios E. Koutelidakis

**Affiliations:** 1 Laboratory of Nutrition and Public Health, Department of Food Science and Nutrition, University of the Aegean, 81400 Myrina, Greece; p.bousdouni@gmail.com (P.B.); olgapap@aegean.gr (O.I.P.);; 2 Department of Food Science and Human Nutrition, Agricultural University of Athens, 11855 Athens, Greece; kapsok@aua.gr; 3 Agia Eleni—Spiliopoulio Pathological Hospital, 11521 Athens, Greecelettastathopoulou@gmail.com (N.S.);; 4 Cardiovascular Research Centre, Department of Cardiovascular Science, University of Leicester, Leicester LE1 7RH, UK; 5 Population, Policy and Practice Research, GOS Institute of Child Health, University College London, London WC1N 1EH, UK; 6 Polykliniki, Olympiako Chorio, 13677 Acharnes, Greece; 7 Laboratory of Applied Microbiology and Biotechnology, Department of Molecular Biology and Genetics, Democritus University of Thrace, 68100 Alexandroupolis, Greecexrysapavl@gmail.com (C.P.); ikourkou@mbg.duth.gr (Y.K.); 8 Laboratory of Organic Chemistry, Department of Chemistry, University of Ioannina, 45110 Ioannina, Greece; 9 QLC, 26442 Patras, Greece

**Keywords:** functional foods, probiotics, oat flakes

## Abstract

*Background and Objectives*: The development of non-dairy probiotic products is a challenge for the food industry, while cereals, as probiotic carriers, provide the means to incorporate probiotics, prebiotics, and fiber into the human diet. The present study investigated the effects of *Lactococcus cremoris* spp. immobilized on oat flakes on blood and urine biomarkers in a randomized placebo-controlled single-blind clinical trial. *Materials and Methods*: Fifty-four eligible participants were randomized into a placebo or probiotic group that consumed 5 g of oat flakes daily for 12 weeks. Blood and urine samples were collected at the baseline, 6 weeks, and 12 weeks to assess the glycemic, lipemic, inflammatory, immunological, and antioxidant biomarkers, as well as the vitamin levels. *Results*: The intervention group exhibited a significant reduction in their hs-CRP levels (*p* = 0.002) and a trend toward decreased IL-6 levels (*p* = 0.035) at week 12 compared to the control group, suggesting a potential anti-inflammatory effect. Additionally, a significant reduction in insulin levels was observed in the probiotic group at week 6, with a clinical trend toward differentiation despite the absence of statistically significant differences between the groups. Furthermore, there were promising results regarding certain biomarkers, such as vitamin B12 and cortisol levels, in the probiotic group. *Conclusions*: The twelve-week consumption of *Lactococcus cremoris* spp. immobilized on oat flakes resulted in improvements in inflammatory, metabolic, and stress-related biomarkers. These results support the examined concept of non-dairy probiotic products, though further research is needed to confirm their efficacy and clarify their underlying mechanisms.

## 1. Introduction

Probiotics are defined as viable microorganisms (bacteria or yeasts) that, when ingested in adequate concentrations, exert various beneficial effects on a host. The most common microorganisms used as probiotics are lactic acid bacteria (LAB), like *Lactococcus*, *Lactobacillus*, *Streptococcus*, *Enterococcus*, and *Bifidobacterium*; however, not all the bacteria can be probiotic, as they need to be strain-specific [1]. To exhibit a beneficial health impact, probiotic microbes should be able to survive in the acidic conditions of the stomach and gastrointestinal tract (GT) of humans, which means being able to withstand the gastric juice and bile salt, survive passage through the upper GT, multiply, colonize, and function in the gut [2].

The health benefits associated with probiotic consumption have been extensively investigated in animal models and human studies; therefore, probiotic applications in foods aim to improve a host’s health and treat different infectious and non-infectious pathologies [3]. The probiotic action of beneficial bacteria can be attributed to various metabolic pathways and is expressed by different mechanisms. Firstly, probiotics play a crucial role in the gut barrier function by causing modulations, which affect the barrier’s robustness and thereby influence disease states [4]. Moreover, the consumption of different probiotic strains may modulate the microbiota, helping to maintain optimal gut health, and preventing/treating chronic inflammatory and immunity-related diseases [5]. Also, probiotics can change the microbiota’s metabolic properties by competing with the absorption of several nutritional substances. Specifically, when health-promoting bacteria are present in the gut, they utilize more nutrients, leaving fewer nutrients for pathogenic bacteria, which may suffer starvation, and thus not survive [6,7].

The ability of probiotics to exert beneficial effects on health is a strain-specific trait [8]. Consequently, in vitro and subsequent in vivo assessments of the probiotic characteristics of wild-type-presumptive probiotic strains, as well as clinical trials, are essential in order to confirm their health-promoting effects. In the present study, the wild-type *Lactococcus cremoris* FBMS_5810 strain, isolated from white mushrooms, was selected. The strain was previously evaluated in vitro for its safety profile and probiotic properties, demonstrating a high cholesterol-assimilation activity and strong adhesion ability to Caco-2 cell lines, an intestinal epithelial cell model [9]. Indeed, high levels of blood cholesterol are considered a significant risk factor for the development of cardiovascular disease, obesity, and other metabolic disorders [10,11]. Thus, the selection of probiotic strains with promising cholesterol-removal activity could be considered an alternative therapeutic approach for hypercholesterolemia. Meanwhile, the ability of probiotics to adhere to and colonize the intestinal mucosa is an important trait by which they exert their beneficial effects [8].

Probiotic delivery has been consistently associated with foods, especially dairy; however, there is an increasing trend toward using probiotics in different food matrixes, regardless of their original source of isolation [12]. In this sense, food ingredients rich in dietary fiber, such as oat flakes, could serve as excellent probiotic vehicles, since there is evidence suggesting the positive impact of dietary fiber on health [13]. Of note, the consumption of oat flakes has been linked to a reduction in cholesterol levels, mainly due to the presence of oat β-glucans that modulate cholesterol metabolism [14]. However, the incorporation of probiotic bacteria into food presents many challenges related to their growth, survival, viability, stability, and functionality during food processing, storage, and consumption, as well as changes in the sensory characteristics of probiotic foods [15]. Probiotic strains exhibit different nutritional and therapeutic functions, due to various factors, such as the genetic make-up of the strain, the amount of probiotics used in a product, the purpose it is used for, and its shelf life [16]. Interest in the development of functional foods consisting of both probiotics and prebiotics have been increasing due to an increased awareness of their health-promoting properties [17]. Although diverse functional lactic acid bacteria have been applied in commercial probiotic fermented foods worldwide, the market for bio-functional products is in continuous need of the diversification of the available products [18,19]. For this purpose, a growing number of scientific studies have focused on the selection of new strains with specific functional properties.

Lactic acid bacteria are widely used in the production of fermented food products, while their metabolic and probiotic characteristics have attracted more attention [20]. Specifically, they have been associated with a variety of products, including short-chain fatty acids, amines, bacteriocins, vitamins, and exopolysaccharides during metabolism [21]. *Lactococcus cremoris* sub sp. *cremoris* was initially identified in fermented milk, while its potential health advantages for humans remain unexplored [22]. In a recent clinical trial, the consumption of a supplement containing *L. cremoris* was found to enhance not just the frequency of bowel movements and the composition of intestinal microbiota, but also various immunological parameters [23]. A study with a similar design examined the effects of fermented milk containing *Lactococcus lactis* subsp. *cremoris* on bowel movements in healthy young Japanese women, and showed increases in the frequency of defecation (days/week and times/week) and stool volume. [24].

Therefore, while there are research data on the effects of *L. cremoris* strains on the gastrointestinal tract, their effects on human health have not been sufficiently studied. The purpose of this interventional study (clinical trial) was to evaluate the effects of the wild-type *L. cremoris* FBMS_5810 strain immobilized on oat flakes, which has previously been evaluated in vivo for its probiotic properties [9], on the blood and urine biomarkers associated with human health. Given the well-established link between elevated cholesterol levels and cardiovascular risk, the strain’s promising in vitro cholesterol-lowering activity provided a strong rationale for its further investigation in a human model, particularly regarding its potential to modulate lipid profiles and related biomarkers.

## 2. Materials and Methods

### 2.1. Preparation of Freeze-Dried L. cremoris Cells Immobilized on Oat Flakes

*L. cremoris* was cultured in 15 L synthetic food-grade medium with the following composition—glucose (20 g/L), yeast extract (25 g/L), KH_2_PO_4_ (2 g/L), CH_3_COONa (6 g/L), MgSO_4_ (0.3 g/L), and MnSO_4_ (0.005 g/L) (pH of 6.5)—and incubated at 30 °C for 24 h in a Fermac 300 bioreactor (Electrolab Biotech Ltd., Tewkesbury, UK) (QLC, Patras, Greece). The freshly grown culture was centrifuged (8500× *g*, 15 min, 4 °C), washed with 1 L sterile ¼ Ringer’s solution (VWR International GmbH, Radnor, PA, USA), and centrifuged again. Then, the cell biomass was resuspended in sterile ¼ Ringer’s solution to the initial culture volume and 7 kg of oat flakes (previously pre-heated at 140 °C for 30 min to avoid contamination) were introduced into the cell suspension. The mixture was left undisturbed for 30 min at ambient temperature and, subsequently, strained and washed with 1 L of sterile ¼ Ringer’s isotonic solution. Then, freeze-drying in a Zirbus (ZIRBUS technology GmbH, Model VaCo 10, Bad Grund, Germany) freeze dryer was carried out, following the method described by Prapa et al. (2025) [25].

### 2.2. Study Design

The present study was a 12-week randomized, placebo-controlled, single-blind clinical trial that was carried out between February and July 2023 at Agia Eleni-Spiliopoulio Pathological Hospital of Athens, Greece. This study’s protocol was approved by the Research Ethics and Ethics Committee of the University of the Aegean (approval 3343/15 February 2022) and it was registered at www.clinicaltrials.gov (ClinicalTrials.gov identifier NCT06293859). All patients were screened after obtaining their written informed consent. In this study, the single-blind design refers to the fact that participants were unaware of whether they were receiving the intervention or the placebo.

### 2.3. Participants 

The present study was advertised to potential volunteers via recruitment flyers that were distributed on the hospital’s premises or posted on social media. Initially, 73 volunteers agreed to participate in this study and underwent in-person screening appointments. Participants were screened using an eligibility checklist containing the inclusion and exclusion criteria. The inclusion criteria were (i) participants aged between 18 and 65 years, (ii) clinically tested fasting plasma glucose less than 100 mg/dL and cholesterol less than 220 mg/dL, and (iii) otherwise healthy. Patients with any of the following exclusion criteria were excluded from this study: (i) body mass index [BMI] higher than 40 kg/m^2^ (morbidly obese); (ii) following a diet plan for weight loss; (iii) on a contraceptive treatment or taking probiotic supplements; (iv) taking medication with an effect on lipemia or glycemia indicators; (v) having any allergies/intolerances to trial ingredients; (vi) pregnant or planning to become pregnant; (vii) breast feeding; (viii) users of illicit drug, those with chronic alcoholism, or total daily alcohol intake more than 50 g per day; (ix) diagnosed with a chronic condition (cancer, active liver disease, severe kidney dysfunction, severe stroke in the last six months, or conditions associated with an increased risk of bleeding) or any other serious medical condition that could affect an individual’s ability to participate in a dietary intervention study; (x) considered unreliable by the researchers, having a shorter life expectancy than the expected duration of this study due to some illness, or if they were in any situation in which, in one of the researcher’s opinions, their participation in this study was not considered safe (e.g., drug addiction, alcohol abuse); and (xi) inability or unwillingness to complete the scheduled follow-up visits. All participants were informed of this study’s aims and procedures, and provided their written informed consent prior to commencing the trial. Compliance with any of the above exclusion criteria during the trial resulted in immediate cessation of participation in this study. 

### 2.4. Intervention

All eligible and consenting participants were assigned a unique code as an identifier and were randomly allocated to receive either probiotic or placebo oat products. The probiotic group received freeze-dried *L. cremoris* FBMS_5810 cells immobilized on oat flakes provided by QLC (Patras, Greece), containing 1.7 × 10^9^ CFU/g (Pavlatou et al. 2025) [9]. Since the recommended probiotic consumption is ≤2 × 10⁹ CFU daily [9]), 2.85 g of freeze-dried *L. cremoris* FBMS_5810 cells immobilized on oat flakes was defined as a suitable amount with which to achieve the daily dose of 2 × 10⁹ CFU, and was mixed with 2.15 g of oat flakes without *L. cremoris* FBMS_5810 cells. The placebo group received oat flakes that were indistinguishable in color, smell, and taste from the flakes with immobilized probiotics. Participants were given written instructions regarding the storage of the product, according to which the product they received had to be stored under refrigerated conditions. All participants were asked to consume 5 g of oats daily as part of a meal (e.g., alongside yogurt intake), provided that the meal was at a temperature below 35 °C and was not acidic. The participants were also instructed to use the same household utensil for daily dosing, which was assessed once using a weighing scale to ensure that it delivered the required 5 g. Moreover, participants were provided with a diary and instructed to record the days on which they consumed the intervention product, as well as the method of consumption (e.g., with yogurt, as part of a specific meal). To assess adherence to this study’s protocol, participants visited the study site every six weeks to return any unused product and obtain a new supply. The returned product was weighed to assess the amount consumed and verify whether consumption was consistent with the entries recorded in the participants’ diaries. Additionally, the participants were asked to maintain their usual dietary habits and physical activity levels throughout the study period and to report any side effects or adverse events they experienced.

### 2.5. Anthropometric and Biochemical Measurements

Data were collected at enrollment (week 0), and at the 6th and the 12th weeks of intervention. Participants’ anthropometric characteristics were measured following standard procedures [26]. Weight (kg) was measured using a suitable body composition monitor (Tanita SC 330 P, Tokyo, Japan), height (cm) was measured using a height meter (Tanita HR 001), and hip and waist circumference (cm) were measured with a measuring tape. To evaluate their medical history, participants were asked to self-complete a medical questionnaire with two demographic questions on their sex and age and three questions on their medical history, namely, “Have you been on any medication therapy in the last three months?”, “Do you take nutritional supplements during the last trimester?”, and “Are you confronted with any clinical illnesses?” At the same time, information was collected about potential gastrointestinal disorders and the weekly frequency of bowel movements. Participants were asked to complete another brief questionnaire to evaluate their nutritional attitudes and general habits. This included a food frequency questionnaire, as well as questions on smoking, physical activity, and alcohol consumption during the preceding 3-month period.

Blood samples were collected by venipuncture at three time points, before intervention and at 6th and 12th weeks. Specifically, 10 mL of blood were collected by a cooperating nursing staff in clot activator tubes for serum collection and in ethylenediaminetetraacetic acid (EDTA). Tubes for each sampling time point were centrifuged in a Thermo Scientific ST16R refrigerated centrifuge (Thermo Fisher Scientific, Waltham, MA, USA) at 3000× *g* and 4 °C for 15 min and 10 min for serum and plasma, respectively. Plasma and serum were then aliquoted and stored at −40 °C until further analysis. Once this study was completed, serum samples (for all time points tested) were analyzed with a COBAS c111 automated biochemical analyzer (Roche, Basel, Switzerland) for high-density lipoprotein cholesterol (HDL-C) and low-density lipoprotein cholesterol (LDL-C), total cholesterol (TC), triglycerides (TGL), high-sensitivity C-reactive protein (hs-CRP), and uric acid (UA). Moreover, insulin (INS), cortisol, immunoglobulin A (Ig-A), interleukin-6 (IL-6), folate, vitamin B12 (VitB12), and vitamin D (VitD) determinations were conducted using a Maglumi 2000 Plus automated immunoassay system (Snibe, Shenzhen, China). The EDTA plasma was used to determine total antioxidant capacity (TAC) at each time point examined, using a ferric-reducing antioxidant power (FRAP) assay, as described by Benzie at al. [27], and for the evaluation of blood glucose levels (biochemical analysis).

Urine samples were delivered by participants to the study investigators on the days of hospital visits, same as in the case of blood samples. The volunteers were given written instructions and consumables for sample collection. To examine urine phosphates, urine samples were acidified with a concentrated HCl 37% to pH < 3 and stored at a refrigerated temperature of 6–8 °C until further analysis. For urine magnesium analysis, samples were acidified to pH 1 with a concentrated HCl 37% and stored at −40 °C until further analysis. Once this study was completed, urine samples were centrifuged in a Thermo Scientific ST16R refrigerated centrifuge (Thermo Fisher Scientific, Waltham, MA, USA) at 1500× *g* and 4 °C for 5 min and were analyzed with a COBAS c111 automated biochemical analyzer (Roche, Basel, Switzerland).

### 2.6. Statistical Analysis

#### 2.6.1. Sample Size

According to sample size calculations, 42 individuals were adequate to detect a significant group interaction effect with an effect size of 0.2, in standardized mean differences, at 5% level with 80% power. To account for 30% drop-out rate, the final sample size was increased to 54 individuals. The sample size was calculated using G*power 3.1 (University of Düsseldorf, Düsseldorf, Germany).

#### 2.6.2. Data Analysis

Descriptive statistics for the concentration of and incremental changes in biomarkers tested are shown as means (Standard Deviation, SD). To detect differences in general characteristics and macronutrients intake between the two study groups, Student’s *t*-test for independent values was used. The significance level was set at 0.05. To detect the effects of intervention, normality was assessed using the Kolmogorov–Smirnov test. Paired-sample *t*-tests were used to detect within-group differences. For comparison of non-parametric categorical variables, Wilcoxon signed-rank test was conducted. Two-way analysis of variance (ANOVA) was used to evaluate between-group changes in variables during this study. The significance level was set at 0.003 after Bonferroni correction. Statistical analysis was carried out using SPSS V21.0 for Windows (IBM Corporation, New York, NY, USA).

## 3. Results

### 3.1. Baseline Characteristics

**Figure 1:**
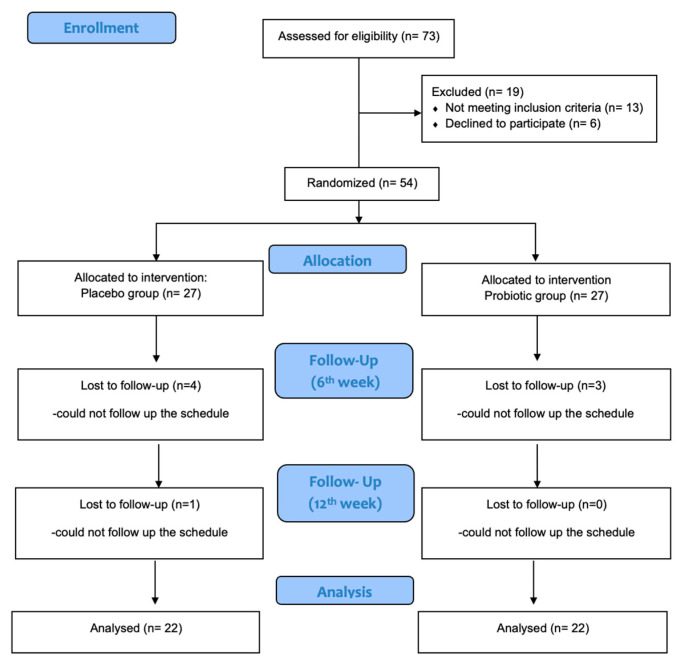
CONSORT schematic of participant recruitment, screening, and assessment [28].

A total of 73 potential participants were initially screened based on the inclusion and exclusion criteria; 13 participants did not meet the conditions for participation in this study, and 6 people decided not to participate after the evaluation process, as they could not meet the schedule that was set. Finally, 54 individuals (n = 15 men and n = 39 women) were eligible and provided their informed consent. The participants were randomly assigned to either the placebo or probiotic groups, and this distribution was unknown to them. This study was completed with 46 volunteers (30% men; probiotic group, n = 24; placebo group, n = 22), as eight people dropped out during the 12-week intervention. The flowchart of this study is presented in Figure 1.

The baseline characteristics of this study’s participants are shown in Table 1. The participants’ gender distribution, mean age, waste-to-hip ratio (WHR), lifestyle habits, total cholesterol, and fasting glucose did not differ between the probiotic and placebo groups at the baseline (all *p*-values > 0.05). Statistically significant differences were found in the participants’ body mass index between the intervention groups, where the average of the probiotic group appeared to be overweight (BMI > 25.0 kg/m^2^), while in the control group, the weight of the volunteers appeared to be at normal levels (BMI < 24.9 kg/m^2^) [29].

### 3.2. Dietary Habits

Comparisons of the dietary intake at the baseline, week 6, and at the end of the trial revealed no significant changes in the dietary macronutrient intake in terms of energy (calories), carbohydrates, proteins, fats, or total dietary fibers within the groups.

### 3.3. Blood Biomarkers

#### 3.3.1. Inflammatory and Immunological Biomarkers

To evaluate the intervention’s effects on inflammation and immune responses, key markers, such as hs-CRP, IL-6, and IgA, were examined. Specifically, the serum hs-CRP levels were significantly reduced in the probiotic group compared with the control group at the 12th week of the intervention (*p* = 0.002). Similarly, the IL-6 levels were significantly reduced at the 12th week in the probiotic group (*p* = 0.035), following a between-group comparison. Among the immune-related biomarkers, IgA was examined, and no statistically significant differences were detected between the groups (*p* = 0.923). The results are presented in detail in Table 2.

#### 3.3.2. Lipemia Biomarkers

To assess the impact of the intervention on the lipid profiles of the participants, key indicators, such as the total, HDL and LDL cholesterol, and triglycerides were examined. According to the results, none of the above indicators presented statistically significant differences between the groups (*p* > 0.003) (Table 2).

#### 3.3.3. Glycemia Biomarkers

The glycemic biomarkers examined were the fasting glucose and insulin. The results showed no statistically significant differences between the intervention group and the control group for either biomarker (*p* > 0.003) (Table 2).

#### 3.3.4. Folate, VitB12, and VitD

The folic acid levels showed a statistically significant decrease in the probiotic group (*p* < 0.001) and the control group (*p* = 0.004) at week 12 of the intervention, with no statistically significant difference between the groups (*p* = 0.944). The vitamin B12 levels were slightly increased in the probiotic group (*p* = 0.036) at week 12 of the intervention, and there were no statistically significant differences between the groups (*p* = 0.762). Finally, the vitamin D levels did not differ significantly between the groups during the intervention (*p* = 0.802).

#### 3.3.5. Cortisol, Uric Acid, and Antioxidant Capacity 

The cortisol levels showed a statistically significant reduction within the probiotic group at the 6th (*p* = 0.003) and 12th (*p* = 0.002) weeks of the intervention, without a statistically significant difference between the intervention and control groups (*p* = 0.814). The uric acid did not differ significantly between the groups, while the total plasma antioxidant capacity increased significantly in the 12th week of the intervention in the probiotic group (*p* = 0.026), without this implying statistically significant differences between the groups (Table 2).

#### 3.3.6. Urine Biomarkers 

The urine biomarkers were used to assess trace minerals, such as magnesium and phosphorus. Specifically, urine magnesium levels were significantly reduced in the probiotic group compared with the control group at the 6th week of the intervention (*p* = 0.01). Regarding the results of the urinary phosphates, no statistically significant changes were noted between the groups (*p* > 0.003). The results are presented in detail in Table 3.

## 4. Discussion

Novel functional foods incorporating probiotics represent a rapidly expanding sector within the food industry, drawing particular attention from the field of nutrition owing to their advantageous impact on human health [30]. Non-dairy probiotic products are of great importance worldwide due to the ongoing increase in vegetarianism, milk’s cholesterol content, and the high prevalence of lactose intolerance in many populations around the world [31]. Non-dairy foods, such as fruits, vegetables, cereals, soy, and meat, known for their abundance in protein, minerals, vitamins, dietary fibers, antioxidants, and various bioactive substances, have been examined for their potential to support the survival and stability of probiotics [32]. Recent scientific evidence has identified β-glucan as a potential prebiotic compound, suggesting its applicability to the development of future health-promoting and therapeutic foods containing probiotics [33]. Oats are a rich source of β-glucan, which act as prebiotics that are selectively fermented by butyrate-producing microorganisms, antioxidant phenolic compounds, dietary lipids, and soluble fiber [34]. The concept of probiotic non-dairy products has been successfully explored through the probiotic fermentation of oats, demonstrating multiple health benefits and functional improvements, including enhanced nutrient bioavailability, antioxidant activity, and applicability in various synbiotic food formulations [35]. In addition to probiotic fermentation, the immobilization of probiotics on oat flakes has also been suggested for evaluation as a promising synbiotic approach, aiming to enhance the survival and colonization of beneficial microbes through the combined effects of prebiotics and probiotics [36].

The present study tested the hypothesis that *Lactococcus cremoris* spp. immobilized on oat flakes may have a positive effect on the blood and urine biomarkers related to chronic diseases and/or nutrient deficiencies in healthy participants. The results for the biomarkers associated with inflammation showed that the hs-CRP was significantly decreased (*p* = 0.002) in the 12th week of the intervention, whereas the IL-6 mean values tended to decline (*p* = 0.035) in the same time frame in the probiotic group. The literature data indicate that the gut microbiota has an influence on the development and maintenance not only of the mucosal, but also the systemic, immune response [37]. Some studies have linked this beneficial effect of probiotics to their potential ability to inhibit the production of pro-inflammatory factors [38]. Several meta-analyses have investigated the effects of probiotics on inflammatory markers in various disease conditions; however, a limited number of studies have examined the effects of probiotics on inflammation in healthy individuals [39]. Among the different strains, a statistically significant probiotic effect was noted on inflammatory biomarkers after 6 weeks of consumption [40], while in a corresponding 8-week study, no differences were noted between groups [41].

Immunoglobulin A (IgA) stands out as the predominant antibody isotype, playing a crucial role in the initial defense against pathogens at mucosal surfaces and contributing to the maintenance of mucosal balance [42]. Secretory IgA plays an important role in protection against infections caused by enteropathogenesis in both human and animal models [43]. Probiotics are known to enhance IgA production as part of their role in supporting mucosal immunity. Intestinal microorganisms, especially during early life, contribute to the development of the acquired immune system and stimulate endogenous IgA production [44]. Oxidative stress is recognized as a major driver of inflammation and can negatively impact immune function; thus, its reduction may help suppress inflammatory responses and support mucosal immunity [45]. In the present study, no statistically significant effect of probiotic consumption on IgA levels was observed. This lack of change may be due to the limited antioxidant capacity of the specific probiotic strain used, which may have been insufficient to reduce oxidative stress and thereby stimulate IgA production. Additionally, the dosage of the probiotic administered may have further influenced these results.

The effect of probiotics on the biomarkers related to the lipid profile has been studied extensively in recent years, and the research results indicate that the estimated time needed to observe more definite results using probiotics in isolation appears to be 6 weeks [46]. According to a meta-analysis that included studies of different probiotic strains in healthy adults, probiotics can significantly reduce serum TC, whereas the same study assumed that the different treatments the intervention groups received, single or multiple strains and low or high doses, did not show significant differences for lowering TC [47]. In the present study, no statistically significant differences were detected between the two groups, whereas in the probiotic group, there was an increase in the total cholesterol levels. Similarly, for the triglyceride levels, no statistically significant differences were noted between the groups; however, there was an increase in triglyceride levels within the probiotic group. The literature suggests that serum cholesterol and triglyceride levels may be more dependent on the degree of adiposity in the volunteers than on the frequency of the consumption of fat, sugar, starch, or alcohol [48]. This explains the increase in cholesterol and triglyceride lipid levels in the probiotic group, while, as shown above, the probiotic group appears to have had a significantly higher BMI compared to the control group. Moreover, individual differences in gut microbiota composition can affect the metabolism of lipids, leading to heterogeneous responses to probiotic intake [49,50]. Finally, the lack of a standardized dietary protocol may have influenced the variation in the measured biomarkers, as the participants’ unrestricted dietary choices could have affected their lipid metabolism and confounded the overall metabolic outcomes.

In conjunction with triglyceride levels, both HDL and LDL cholesterol levels serve as risk factors for cardiovascular disease. The effect of probiotics on HDL and LDL cholesterol levels is highly heterogeneous; however, it has been observed that in individuals with higher metabolic risk, the effect of probiotics on reducing LDL and increasing HDL cholesterol is higher [51,52,53]. In clinical trials employing a similar design, there were no discernible metabolic variations observed in the aforementioned biomarkers among the healthy participants [54,55]. In the present study, no statistically significant effect of probiotic consumption on HDL- and LDL- cholesterol levels was observed. This may be attributed to the health status of the participants, as they were not individuals with a higher metabolic risk. Moreover, the lack of change in the examined biomarkers may be strain-related.

Abnormal glucose metabolism is causally related to a greater risk of several chronic disorders, including dyslipidemia and cardiovascular diseases, and dietary constituents and supplements have been proposed as improving glycemic control [56]. Several trials have suggested that probiotic consumption may prevent or reduce elevated blood glucose levels, while the glucose-lowering effects of *Lactobacillus* and *Bifidobacteria* have been investigated in several human studies [57]. In the present study, examining the effect of *Lactococcus cremoris* spp., no statistically significant differences were found between the two groups. In parallel with glucose metabolism, the impact of probiotics on insulin levels is an area of ongoing research, and several studies have suggested that probiotic supplementation may improve insulin metabolism [58]. According to the results of the present study, a significant reduction in insulin levels was detected within the probiotic group in the 6th week of the intervention, which returned to baseline values during the 12th week of the intervention. Although no statistically significant differences were found between the probiotic and control groups for most of the parameters, a clinical tendency toward differentiation was observed (*p* = 0.028).

A further examination of the biomarkers associated with cardiovascular diseases, such as serum cortisol, uric acid levels, and plasma total antioxidant capacity (TAC), was performed. Cortisol, which is synthesized from cholesterol, is the main glucocorticoid in the zona fasciculata of the human adrenal cortex [59], and it was recently recognized that cortisol may be involved in a number of forms of hypertension [60] and other cardiovascular risk factors, such as hyperinsulinemia, hyperglycemia, insulin resistance, and dyslipidemia [61]. Data on the effects of probiotic strains on cortisol, as well as the metabolic mechanisms that bring about possible changes, are limited. A symbiotic consisting of *Lactobacillus* spp., *Bifidobacterium* spp., and prebiotics reduced serum cortisol levels after 12 weeks of consumption [62], whereas a study with a similar intervention product observed a reduction in urinary cortisol over 8 weeks [63]. However, a study of functional yogurt containing *Lactobacillus* spp. and *Bifidobacterium* spp. observed no statistically significant differences in cortisol secretion over a 12-week period [64]. In the present study, a statistically significant reduction in cortisol secretion was identified during the 6th week of the intervention in the probiotic group, which was maintained at the 12th week of the intervention. However, no statistically significant differences were found between the two groups.

While the correlation between serum uric acid and cardiovascular disease has long been acknowledged, it remains inconclusive whether serum uric acid acts as a causative agent in cardiovascular disease or merely represents a risk factor strongly linked to established cardiovascular risk factors, such as hypertension and dyslipidemia [65]. Considerable clinical and epidemiological evidence supports the claim that probiotics reduce serum uric acid levels, through three often overlooked mechanisms: (1) metabolizing purines into compounds distinct from uric acid; (2) diminishing the activity of xanthine oxidase (a liver enzyme associated with uric acid production); and (3) enhancing the expression of uric acid transporter proteins to facilitate the excretion of uric acid [66]. In an 8-week randomized trial, the daily consumption of yogurt enriched with *Lactobacillus acidophilus* and *Bifidobacterium lactis* significantly reduced serum uric acid levels in patients with metabolic syndrome [67]. In a same-design study, a yogurt beverage enriched with *Lactobacillus gasseri* reduced serum uric acid levels in patients with hypouricemia [68]. The present study detected no difference between the groups in their serum uric acid levels, probably because healthy volunteers with values within normal limits participated.

In recent years, the number of studies, both in vitro and in vivo, related to the antioxidant properties of probiotics has significantly increased, while the development of probiotics that exert antioxidant activity and counteract oxidative stress has emerged as a novel approach to reduce oxidative stress [69]. Oxidative stress is also a key factor in the pathogenesis of CVD (e.g., atherosclerosis), and a disorder of pro-oxidant/antioxidant balance and the domination of pro-oxidative reactions may lead to oxidative stress in the nervous system, which may affect brain development and function [70]. In a seven-week study, a synbiotic capsule containing *Lactobacillus casei* with inulin was found to be an effective compound that protects the human body from oxidative stress damage and increases the total antioxidant plasma capacity [71]. However, in the present study, no statistically significant differences were found between the two groups.

Given the inability of humans to internally synthesize most vitamins, reliance on exogenous sources is necessary, and the utilization of microorganisms capable of producing vitamins presents a potentially more natural and consumer-friendly alternative to fortification with chemically synthesized pseudo-vitamins [72]. Folate is a B-group vitamin involved in many metabolic pathways, such as energy usage, nucleic acid synthesis, and one-carbon metabolism. Recent findings have linked folate levels to a reduction in neural tube defects, coronary heart diseases, and cancer [73]. LAB have been reported to be folate producers; however, the ability of microbial cultures to produce or utilize folate varies considerably and is a strain-dependent trait [74]. The research indicates that substantial folate synthesis within the human gastrointestinal tract could be clinically relevant if bioavailable, with direct in vivo evidence demonstrating the absorption of bacterially synthesized folate across an intact large intestine and its incorporation into tissues [75]. Vitamin B12, like folate, is associated with preventing chronic diseases associated with aging through the methylation of homocysteine [76]. Vitamin B12, otherwise known as cobalamin, has the most complex structure of all the vitamins synthesized by bacteria, requiring about 30 genes for its biosynthesis [77]. In addition to the production of vitamin B12 by some probiotic bacteria, probiotics might improve vitamin B12 status by altering the composition of the gut microbiome and reducing the abundance of intestinal bacteria involved in B12 catabolism [78]. Improvements in nutrient status following the administration of certain probiotics have been noted for B-group vitamins (folate and B12) [79,80,81]; however, clinical trials assessing probiotic supplementation have yielded varying outcomes across diverse micronutrients. In the present study, the effect of the *Lactococcus cremoris* strain on folate levels in healthy volunteers was assessed, revealing no statistically significant differences between the intervention groups. This is likely attributable to the inability of the strain to produce folate, which may be due to the absence or downregulation of key genes in the folate biosynthesis pathway. Specifically, folate production by LAB often depends on the availability of precursors, such as pABA, and the ability of the strain to uptake and utilize them effectively [74]. Furthermore, vitamin B12 levels were significantly increased within the probiotic group; however, this change was not detected between the groups, which suggests a possible strain-specific or dosage role in modulating B12 metabolism or uptake.

Vitamin D, a fat-soluble vitamin, is essential for the development and maintenance of bone tissue, as well as for the normal homeostasis of calcium and phosphorus and the normal functioning of the immune system [82]. Implicated in the onset of various chronic endocrine and metabolic disorders, vitamin D deficiency is associated with a decreased risk of cardiovascular diseases, diabetes, and metabolic syndrome, as indicated by meta-analyses that have emphasized the importance of maintaining sufficient vitamin D concentrations in adults [83]. Evidence from a clinical trial suggested the role of the probiotic bacteria *Lactobacillus reuteri* in increasing vitamin D levels [84], while further data on the effect of probiotic ingredients on vitamin levels are needed. The proposed mechanisms underlying the observed increase in vitamin D levels following probiotic supplementation include enhanced intestinal absorption mediated by improved gut barrier integrity, the modulation of luminal pH and ion concentrations, and the potential upregulation of key enzymes involved in vitamin D metabolism [84]. In the present study, no significant changes in vitamin D levels were observed between the groups, which may be attributed to the specific characteristics of *Lactococcus cremoris*. This strain may lack the mechanisms required to modulate vitamin D metabolism, potentially explaining the absence of observed effects. Additionally, factors, such as individual dietary intake and variations in sunlight exposure, may have contributed to the results, potentially masking any subtle effects of the probiotic on vitamin D levels.

Moving on to the urine biomarkers, the phosphate and magnesium levels were examined. Urine magnesium (Mg) levels indicate the Mg content in the body, while Mg-deficient volunteers tend to retain a greater proportion of a Mg load and, consequently, excrete less Mg in their urine than individuals with normal levels do [85]. Probiotics can stimulate the quantitative or qualitative composition of the intestinal microflora to improve and increase magnesium bioavailability [86]. According to in vivo results, multistrain probiotic consumption tends to affect magnesium levels in some organs through absorption and distribution processes in the organism. The present study detected a significant reduction in urine magnesium levels at the 6th week of the intervention for the probiotic group, which indicates that magnesium was incorporated into the cells due to its necessity. In the case of urine phosphorus, the data remain limited, while a study examining the effects of probiotics on urine phosphorus absorption did not detect statistically significant differences [87]. These data are consistent with the results of the present study, according to which no statistically significant differences in urine phosphorus absorption were detected between the two groups.

Also, it should be stated that the present study has some limitations. First, a 12- week dietary intervention might not be sufficient to change the gut microbiota composition and blood markers in healthy subjects due to an unhealthy lifestyle. However, prolonging the duration of the study could potentially impact compliance, thereby negating any potential benefits. Second, the participants were not provided with a dedicated dispenser for the intervention product, but instead were instructed to use household utensils for measuring the prescribed dose, a method that may have introduced variability into the measurements. A further limitation of this study is the exclusive use of directly measured biochemical indicators obtained through enzymatic analyses, without incorporating additional clinically relevant markers that are indirectly calculated or associated with chronic disease risk, potentially limiting the comprehensiveness of the metabolic assessment. Despite the above, this study was significantly and adequately powered to investigate the efficacy of probiotics immobilized on oat flakes on blood and urine biomarkers.

## 5. Conclusions

This randomized, placebo-controlled, single-blind clinical trial demonstrated that the daily consumption of Lactococcus cremoris spp. immobilized on oat flakes for 12 weeks may exert beneficial effects on various health-related biomarkers. Significant reductions in hs-CRP levels and a trend toward decreased IL-6 levels suggest the potential anti-inflammatory properties of the intervention. Additionally, favorable changes in insulin, vitamin B12, and cortisol levels suggest metabolic and stress-related benefits. These findings support the potential of cereal-based non-dairy probiotic products as novel functional foods. However, considering the multifactorial nature of host–microbiota interactions and the methodological heterogeneity in biomarker assessment, further well-powered mechanistic studies are warranted to substantiate these results and elucidate the underlying pathways.

## Figures and Tables

**Table 1:** General characteristics of participants at baseline.

Variables	Probiotic Group (n = 24)	Control Group (n = 22)	*p*-Value ^a^
Female (%)	70.8	68.2	*p* = 0.847
Age (years)	36.6 (13.9)	30.3 (10.2)	*p* = 0.301
Height (m)	1.68 (0.1)	1.66 (0.1)	*p* = 0.436
Body mass index (kg/m^2^)	26.9 (5.5)	23.3 (3.5)	*p* = 0.021
Waist-to-hip ratio	0.82 (0.1)	0.80 (0.1)	*p* = 0.300
Smoking (%)	25.0	18.2	*p* = 0.580
Physical activity (%)			*p* = 0.919
*High*	29.2	40.9	
*Regular*	25.0	13.6	
Cholesterol (mg/dL)	171.0 (24.4)	172.1 (25.8)	*p* = 0.657
Glucose (mg/dL)	79.8 (11.5)	79.6 (9.7)	*p* = 0.586

Data are presented as mean (SD) unless otherwise indicated. ^a^: by independent *t*-tests.

**Table 2:** Serum and plasma biomarkers for each intervention group.

	Probiotic Group (n = 24)			Placebo Group (n = 22)			*p* ^b^
Total Cholesterol	Mean (SD)	Δ from Baseline	*p* ^a^	Mean (SD)	Change	*p* ^a^	
*1st week*	171.0 (24.4)			172.1 (25.8)			0.673
*6th week*	190.2 (38.2)	19.2 (44.0)	0.039	176.1 (40.0)	4.0 (30.4)	0.548	
*12th week*	175.8 (28.9)	4.8 (28.3)	0.040	179.3 (49.7)	7.1 (33.2)	0.325	
**LDL**							
*1st week*	86.2 (21.8)			86.9 (23.4)			0.468
*6th week*	91.3 (35.9)	5.2 (28.0)	0.373	80.5 (29.5)	−6.4 (18.0)	0.108	
*12th week*	91.7 (25.8)	5.8 (14.0)	0.064	92.3 (34.8)	5.4 (17.7)	0.168	
**HDL**							
*1st week*	53.5 (13.2)			54.1 (9.8)			0.313
*6th week*	51.6 (16.1)	−1.9 (14.9)	0.544	53.6 (13.2)	−0.5 (13.0)	0.858	
*12th week*	54.63 (12.0)	1.1 (9.3)	0.560	58.5 (16.0)	4.4 (11.4)	0.086	
**TRGL**							
*1st week*	73.7 (30.4)			83.5 (64.5)			0.719
*6th week*	77.1 (37.1)	3.4 (28.4)	0.530	79.7 (52.9)	−3.9 (36.5)	0.615	
*12th week*	82.4 (34.6)	8.7 (19.1)	0.04	80.0 (43.9)	−3.5 (29.6)	0.685	
**GLU**							
*1st week*	79.8 (11.5)			79.6 (9.7)			0.660
*6th week*	90.3 (19.4)	10.5 (21.5)	0.025	86.3 (16.3)	6.7 (12.4)	0.019	
*12th week*	93.1 (10.5)	13.3 (10.4)	<0.001	93.9 (11.2)	14.3 (10.6)	<0.001	
**INS**							
*1st week*	9.5 (3.4)			7.7 (3.4)			0.028
*6th week*	7.8 (2.9)	−1.7 (1.9)	<0.001	8.2 (4.9)	0.5 (3.7)	0.528	
*12th week*	11.2 (5.8)	+1.7 (3.7)	0.042	7.8 (3.0)	0.1 (2.4)	0.908	
**UA**							
*1st week*	4.5 (1.4)			4.5 (1.1)			0.888
*6th week*	4.0 (1.8)	−0.5 (0.9)	0.008	4.1 (1.4)	−0.4 (0.9)	0.025	
*12th week*	4.6 (1.2)	0.1 (0.6)	0.495	4.7 (1.2)	0.2 (1.0)	0.341	
**Cortisol**							
*1st week*	165.7 (64.8)			154.6 (50.1)			0.814
*6th week*	131.0 (44.9)	−34.8 (50.6)	0.003	139.8 (52.6)	14.8 (36.4)	0.070	
*12th week*	137.5 (48.4)	−28.2 (38.4)	0.002	132.7 (61.3)	22.0 (58.1)	0.091	
**hs-CRP**							
*1st week*	20.2 (21.7)			9.1 (0.0)			0.002
*6th week*	21.6 (33.0)	1.4 (32.0)	0.179	7.9 (11.0)	−1.2 (10.2)	0.067	
*12th week*	16.8 (20.8)	−3.4 (16.3)	0.376	10.3 (12.0)	1.2 (12.5)	0.615	
**IgA**							
*1st week*	2265.0 (842.3)			2188.5 (1179.0)			0.923
*6th week*	2278.2 (752.9)	13.2 (608.1)	0.998	2515.4 (1304.1)	327.9 (1266.7)	0.263	
*12th week*	2178.8 (716.2)	−86.2 (377.1)	0.280	2213.8 (952.1)	25.4 (780.4)	0.880	
**IL-6**							
*1st week*	4.3 (2.6)			3.0 (1.8)			0.035
*6th week*	2.5 (2.0)	−1.8 (2.6)	0.002	1.5 (0.9)	−1.6 (2.2)	0.004	
*12th week*	2.9 (1.9)	−1.3 (2.9)	0.03	2.9 (2.5)	−0.2 (3.4)	0.816	
**Folate**							
*1st week*	8.3 (3.7)			7.7 (3.7)			0.944
*6th week*	7.2 (3.3)	−1.1 (2.1)	0.012	7.7 (5.7)	−0.1 (1.9)	0.860	
*12th week*	5.9 (3.6)	−2.2 (2.2)	<0.001	5.5 (3.8)	−2.2 (3.2)	0.004	
**VitB12**							
*1st week*	468.7 (115.5)			488.8 (187.5)			0.762
*6th week*	502.3 (103.3)	33.6 (83.4)	0.061	511.6 (169.2)	22.8 (120.4)	0.385	
*12th week*	522.7 (114.4)	54.0 (118.7)	0.036	506.8 (121.3)	18.0 (93.6)	0.378	
**VitD**							
*1st week*	23.5 (9.1)			23.3 (8.2)			0.802
*6th week*	22.3 (8.5)	−1.2 (3.0)	0.057	24.0 (8.3)	0.7 (4.1)	0.449	
*12th week*	25.9 (6.2)	2.4 (7.7)	0.138	25.1 (5.2)	1.7 (6.7)	0.239	
**TAC**							
*1st week*	0.8 (0.2)			0.8 (0.2)			
*6th week*	0.9 (0.2)	0.04 (0.1)	0.137	0.8 (0.2)	0.03 (0.1)	0.241	0.391
*12th week*	0.9 (0.2)	0.06 (0.1)	0.026	0.9 (0.2)	0.05 (0.1)	0.085	

*p* ^a^ indicates differences within groups; *p* ^b^ indicates differences between groups.

**Table 3:** Urine biomarkers for each intervention group.

	Probiotic Group (n = 24)			Placebo Group (n = 22)			*p* ^b^
Urine Magnesium	Mean (SD)	Δ from Baseline	*p* ^a^	Mean (SD)	Change	*p* ^a^	
*1st week*	11.0 (5.7)			9.8 (7.7)			0.585
*6th week*	7.9 (3.9)	−3.05 (5.6)	0.013	8.2 (7.4)	−1.7 (9.7)	0.433	
*12th week*	10.4 (6.6)	−0.6 (8.2)	0.713	9.0 (6.2)	−0.9 (6.4)	0.529	
**Urine Phosphorus**							
*1st week*	103.7 (54.0)			98.8 (44.8)			0.933
*6th week*	105.0 (45.4)	1.3 (50.3)	0.902	91.0 (41.3)	−7.7 (54.8)	0.516	
*12th week*	106.1 (66.3)	2.4 (50.6)	0.817	120.9 (59.3)	22.1 (73.8)	0.174	

*p* ^a^ indicates differences within groups; *p* ^b^ indicates differences between groups.

## Data Availability

The data are available upon request.
